# Blue Crab (*Callinectes sapidus*) Haemolymph as a Potential Reservoir of Mesophilic *Shewanella* Species

**DOI:** 10.3390/ani15121731

**Published:** 2025-06-11

**Authors:** Giuseppe Esposito, Fabio Bondavalli, Matteo Riccardo Di Nicola, Paolo Pastorino, Sonia Scala, Martina Gini, Giulia Milanese, Edoardo Turolla, Alessandra Maganza, Simona Sciuto, Domenico Meloni, Rita Melillo, Pierluigi Acutis, Elena Bozzetta, Sebastiano Virgilio, Caterina Faggio, Silvia Colussi, Marino Prearo

**Affiliations:** 1 Istituto Zooprofilattico Sperimentale del Piemonte, Liguria e Valle d’Aosta, 10154 Turin, Italy; giuseppe.esposito@izsplv.it (G.E.); paolo.pastorino@izsplv.it (P.P.); sonia.scala@izsplv.it (S.S.); martina.gini@izsplv.it (M.G.); giulia.milanese@izsplv.it (G.M.); alessandra.maganza@izsplv.it (A.M.); simona.sciuto@izsplv.it (S.S.); pierluigi.acutis@izsplv.it (P.A.); elena.bozzetta@izsplv.it (E.B.); silvia.colussi@izsplv.it (S.C.); marino.prearo@izsplv.it (M.P.); 2 Wildlife Health Ghent, Faculty of Veterinary Medicine, Ghent University, Salisburylaan 133, 9820 Merelbeke, Belgium; 3 Istituto Delta—Ecologia Applicata, 44124 Ferrara, Italy; veliger@istitutodelta.it; 4 Dipartimento di Medicina Veterinaria, Università degli Studi di Sassari, 07100 Sassari, Italy; dmeloni@uniss.it (D.M.); sebastiano.virgilio@izs-sardegna.it (S.V.); 5 Istituto Zooprofilattico Sperimentale della Sardegna, 07100 Sassari, Italy; rita.melillo@izs-sardegna.it; 6 Dipartimento di Scienze Chimiche, Biologiche, Farmaceutiche e Ambientali, Università degli Studi di Messina, 98121 Messina, Italy; caterina.faggio@unime.it; 7 Dipartimento di Biotecnologie Marine Ecosostenibili, Stazione Zoologica Anton Dohrn, 80121 Naples, Italy

**Keywords:** aquatic ecosystems, Adriatic Sea, antibiotic susceptibility, blue crab pathogens, crustacean, invasive species, Italy, marine microbiology, Mediterranean

## Abstract

The blue crab (*Callinectes sapidus*) is an invasive alien species in the Mediterranean Sea, posing threats to biodiversity, fisheries, and aquaculture. Climate change has worsened these challenges, influencing the distribution of bacterial species, including *Shewanella* species, which are sensitive to changes in temperature and salinity. In this study, 300 blue crabs were sampled between June and October 2024 from the Sacca di Goro (Northern Adriatic Sea, Italy) to investigate the prevalence of *Shewanella* species in their haemolymph. The prevalence was found to be 7% (21/300), with species such as *S. mesophila*, *S. algae*, *S. cowelliana*, and *S. baltica* identified, particularly in the months of September and October. Molecular techniques, including MALDI-TOF MS and *rpoB* gene amplification, were used to identify isolates. Antibiotic susceptibility testing (AST) revealed a trend of resistance to beta-lactam antibiotics. A network analysis was also conducted to examine the global trends of *Shewanella* research in relation to humans, animals, and the marine environment. While proper cooking eliminates the risk to consumers, handling without personal protective equipment can increase exposure, particularly for vulnerable individuals such as those who are elderly or immunocompromised. Mild symptoms are observed in children. Further studies, particularly with a One Health approach, are crucial to better understand the transmission dynamics and evolving antibiotic resistance of *Shewanella* species.

## Simple Summary

This study was conducted to understand whether blue crabs living in the Sacca di Goro lagoon (northeast Italy) could be carriers of bacteria that may pose a risk to people or the environment. Some of these crabs had a bacterium called *Shewanella* in their haemolymph, especially during the warmer months of September and October. These bacteria are usually found in the sea but can occasionally cause infections in humans. Some were also resistant to certain antibiotics, which makes treatment more difficult in the event of an infection. Our findings suggest that vulnerable people, such as those with weakened immune systems, should be cautious when handling raw crabs and that crabs should always be properly cooked before consumption. These results underscore the importance of continued research into bacterial transmission dynamics at the human–animal–environment interface, with implications for both public and marine health.

## 1. Introduction

In the Mediterranean Sea, the blue crab, *Callinectes sapidus* Rathbun 1896 (Brachyura, Portunidae), is an invasive species threatening biodiversity, fisheries, and aquaculture, spreading since 1948 via ballast waters [1,2,3]. Its spread in the western Mediterranean Sea and its outbreak in the Northern Adriatic Sea in 2023 caused severe damage to fisheries and shellfish industries, particularly affecting burrowing bivalves, such as the Japanese carpet shell (*Ruditapes philippinarum*), reducing catches and revenues [4,5].

However, crustaceans are important model organisms for research on marine ecology and pathogen–host interactions, with bacterial diseases in crabs being studied since the 1970s [6]. Their haemolymph, crucial for immunity, can host bacteria, influenced by environmental stressors, with counts of up to 6.7 × 10^5^ CFU mL^−1^ [7,8]. Blue crabs are constantly exposed to pathogens in seawater and sediments, carrying bacterial loads of up to 10⁹ CFU mL^−1^ [9]. Diseases in crustaceans pose health risks to both crabs and humans, with concerns about contaminants and improper cooking [10,11,12].

Nowadays, climate change is a major challenge, altering ecosystems and threatening biodiversity [13,14]. Rising temperatures and salinity levels, as projected under climate change scenarios, may lead to an increase in bacterial abundance and contamination levels in seafood, posing potential risks to food safety and public health [12]. Among the bacterial taxa influenced by these environmental changes, *Shewanella* MacDonell and Colwell 1986 species are particularly affected, as their spread is closely linked to variations in temperature and salinity. In particular, these bacteria are also known to thrive in extreme conditions, such as low-temperature, high-pressure, and elevated-salinity environments, further highlighting their adaptability [15].

In addition to their environmental resilience, *Shewanella* species are increasingly recognised as emerging multidrug-resistant pathogens. They have been found to carry β-lactam and quinolone resistance genes located on mobile genetic elements, including plasmids and integrons, posing significant challenges for antibiotic efficacy and treatment [16,17].

Infections caused by *Shewanella* species generally occur in warm climates or during unusually warm summers in temperate regions, where elevated temperatures create favourable conditions for their proliferation [18].

Thus, the Mediterranean is an environmental hotspot, as it is warming 20% faster than the global average, putting additional stress on ecosystems, economies, and societies [19]. Coastal areas face higher risks of flooding, erosion, and salinisation, with water temperatures expected to rise by +1.8 °C to +3.5 °C by 2100, especially in Spain and the Eastern Mediterranean [19].

*Shewanella* was initially isolated in 1931 from putrefied butter and was named variously as *Achromobacter putrefaciens*, *Pseudomonas putrefaciens*, and *Alteromonas putrefaciens*, before being classified in its current genus, which now includes more than 70 identified species [20,21,22]. *Shewanella* species are Gram-negative, oxidase-positive, facultatively anaerobic bacteria with a single polar flagellum that are widely recognised for their presence in marine ecosystems worldwide [23,24] (Table S1). Although they are primarily saprophytic and opportunistic pathogens, certain species like *Shewanella algae*, *Shewanella putrefaciens*, and *Shewanella xiamenensis* have been rarely implicated in a range of clinical infections [16,18,24,25,26,27], including skin and soft tissue conditions, E.N.T.-related diseases, abdominal and chest infections, bacteraemia, and even cardiovascular and neurological complications. These infections have raised significant global health concerns [28].

Also, *Shewanella* species have been often linked to diarrhoeal diseases, with isolates obtained from food poisoning cases and patients with diarrhoea [29,30,31]. Traditional identification methods are challenging due to phenotypic similarity among species [32] and limitations in 16S rRNA gene analysis [33]. Thus, advanced approaches like multilocus sequence typing [34,35] and whole-genome sequencing enhance the understanding of their taxonomy, drug resistance, and virulence [36].

Such bacteria, including *S. algae*, have been isolated from haemolymph samples of blue crab in the Goro lagoon (Northern Adriatic Sea, Italy). The aim of this preliminary study is to highlight the presence of these microorganisms at the species level in the haemolymph of an invasive alien species, which has seen significant spread since 2023 [37]. The enumeration of CFUs per millilitre of haemolymph was not performed, as the primary objective of this study was not to quantify bacterial load but rather to characterise the presence of specific bacterial groups. Given that blue crabs were not infected and the bacterial community detected likely represents their normal microbiome, the focus was placed on identifying taxa rather than absolute abundances. Moreover, variations in haemolymph volume and bacterial distribution could introduce biases in CFU quantification, making qualitative approaches more suitable for addressing the research question.

## 2. Materials and Methods

### 2.1. Shewanella Isolation: Sampling Site and Procedures

**Figure 1:**
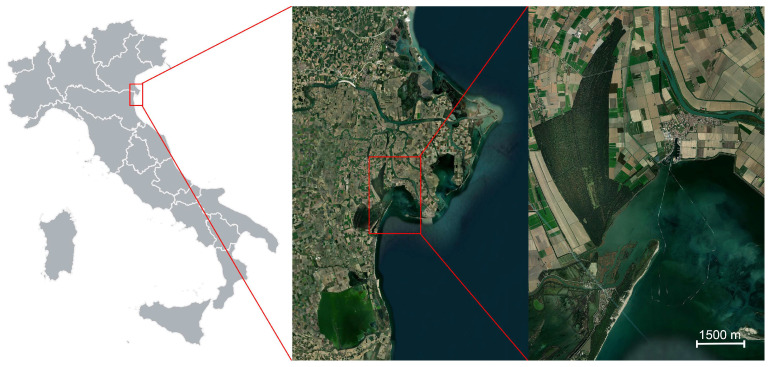
Sampling site (Goro, Emilia-Romagna, Italy).

Between June and October 2024, sampling was carried out at a fixed point within the Sacca di Goro, Italy (Northern Adriatic Sea; 44°48′57″ N, 12°19′09″ E; Figure 1).

The sampling months were selected to ensure representation of the biological cycle, considering that after spring, smaller size classes become less available, requiring a targeted approach for meaningful comparison. A total of 300 blue crabs were sampled using fish-baited traps set at depths between −0.5 and −1.0 m, with 60 crabs collected each month. The crabs were then divided into two size categories: small (n = 30; ≤99 mm) and large (n = 30; ≥100 mm), and classified by sex. Morphological measurements were taken with a digital calliper (0.1 mm resolution), and body wet weights were recorded using a VEVOR Digital Balance (0.01 g precision).

Following standard fishing and storage procedures, *Callinectes sapidus*, a species not included among those protected under Legislative Decree No. 26 of 4 March 2014 [38] (implementing Directive 2010/63/EU [39] on the protection of animals used for scientific purposes), underwent a pre-cooling phase (approximately 30 min) carried out by fishermen at temperatures between 4 °C and 8 °C. After this phase, haemolymph was collected on site under sterile conditions to minimise any impact on haemolymph viscosity and ensure sample integrity. A 1 mL sterile syringe with a 26Ga needle was used to withdraw haemolymph from the leg joints or between the carapace and the abdomen, after swabbing external surfaces with 70% ethanol to prevent contamination. Since crustaceans are not covered by the cited decree, no specific ethical authorisation was required. Also, given that the blue crab is listed as an invasive alien species (IAS) under Regulation (EU) No. 1143/2014 [40] and Legislative Decree No. 230 of 15 December 2017 [41], handling practices were conducted in accordance with regulations aimed at preventing the spread of IAS. After sampling, the crabs were refrigerated at −4 °C for 24 h and subsequently frozen at −20 °C, following standard post-harvest handling procedures and respecting animal welfare standards.

After haemolymph collection, two drops of each sample were streaked onto Marine Agar 2216 (Zobell Marine Agar) plates (Sigma-Aldrich, St. Louis, MO, USA) using sterile loops to isolate heterotrophic marine bacteria. The plates were incubated at 22 ± 2 °C for 72 h [42], with daily checks. Each sample was also streaked onto laboratory-prepared thiosulphate–citrate–bile salts–sucrose (TCBS) agar and CHROMagar™ *Vibrio* (CHROMagar Microbiology, Paris, France). Dominant colonies and presumptive *Shewanella* colonies, characterised by specific colony morphology on both media, were subcultured and identified via MALDI-TOF MS. In cases where species-level identification was inconclusive for the class of Gammaproteobacteria, molecular analysis was performed.

### 2.2. Seawater Parameter Collection

The main physico-chemical variables of the seawater, including temperature (°C), salinity (g/L), and pH were directly measured during each sampling event using a multi-parameter probe (HI98194, HANNA^®^ instruments, Padova, Italy) at a depth of −0.5 m. These in situ measurements allowed for an accurate characterisation of the environmental conditions at the time of sampling, providing context for the occurrence and distribution of bacterial isolates (Figure S1).

### 2.3. Antimicrobial Susceptibility Testing

Antibiotic susceptibility testing (AST) was conducted using the disc diffusion method (Kirby–Bauer test) to evaluate bacterial resistance. Since no specific breakpoints are available for *Shewanella*, the inhibition zones were measured and classified as susceptible (S), intermediate (I), or resistant (R) according to the Clinical and Laboratory Standards Institute (CLSI) guidelines. Results were interpreted based on the criteria outlined in VET03 [43] and VET04 [44] for aquatic animal pathogens. The study used antimicrobial susceptibility discs (Biolab Inc., Budapest, Hungary) for a range of antibiotics, including amoxicillin (AX-25 µg), ampicillin (AM-10 µg), enrofloxacin (ENR-5 µg), florfenicol (FFC-30 µg), flumequine (UB-30 µg), oxolinic acid (OA-10 µg), oxytetracycline (T-30 µg), trimethoprim/sulphamethoxazole (SXT-1.25/23.75 µg), tetracycline (TE-30 µg), and thiamphenicol (TP-30 µg). Bacterial isolates were streaked onto Mueller–Hinton agar supplemented with 2% NaCl in three directions, rotating the plate 60° after each streak. After allowing the surface to dry for 15–20 min, antimicrobial discs were placed on the agar using sterile forceps. The plates were then incubated overnight at 22 ± 2 °C.

### 2.4. Genetic Characterisation and Identification

DNA extraction was performed using the boiling and freeze–thawing method [45]. To identify bacteria species, the RNA polymerase beta subunit (*rpoB*) gene was amplified using specific primers developed by Ki et al. [46]. The amplicons were separated on a 2% agarose gel stained with GelGreen (Biotium, Fremont, CA, USA) and visualised under UV light, with a 50–2000 kb ladder (Amplisize Molecular Ruler, Bio-Rad, Hercules, CA, USA) used as the molecular marker. Amplicons were purified using the ExtractMe DNA Clean-Up and Gel-Out Kit (Blirt, Gdańsk, Poland), and bidirectional sequencing was performed using Big Dye 3.1 (Applied Biosystems, Waltham, MA, USA) chemistry and the same primers. Sequencing products were purified with the Dye Ex 2.0 Spin Kit (QIAGEN, Hilden, Germany) and analysed on an SeqStudio Genetic Analyzer (Applied Biosystems, Waltham, MA, USA). DNA sequences were assembled into contigs using Lasergene Software 18.0.1 (DNASTAR, Madison, WI, USA) and compared to GenBank sequences via BLAST+ 2.16.0 (NCBI, Bethesda, MD, USA).

### 2.5. PubMed Database Analysis of Shewanella Species

The research was conducted using the “Advanced Search Builder” function of PubMed, one of the leading biomedical databases. The keywords used for the investigation were (*Shewanella*) AND (Human) AND (Marine), with studies filtered for English language and only focusing specifically on marine coastal environments to align with the ecological habitat of the blue crab. These specific keywords were chosen to avoid narrowing the number of relevant studies and to focus on the primary species of interest in humans, linked to the coastal marine environment. This approach highlighted an increase in clinical infection cases caused by these species, affecting both aquatic organisms and humans. The dataset was saved in PubMed format (.txt), and uploaded to VOSviewer (v. 1.6.20) for analysis [47]. A co-occurrence analysis was conducted to assess the relationships between items by examining the frequency with which they appeared together in the same documents. To achieve a more consistent perspective less influenced by variations in terminology and to enhance data aggregation and comparability, “MeSH keywords” were used as the unit of analysis. Keywords with a minimum occurrence threshold of 5 were included in the analysis.

### 2.6. Statistical Analysis

Statistical analysis was conducted using R software (version 4.3.2; R Core Team, 2024). Also, to minimise any unnecessary use of animals, the sample size was carefully estimated using G*Power software (version 3.1.9.7) to ensure statistical validity while avoiding excessive sampling.

The normality of the data was evaluated with the Shapiro–Wilk test (shapiro.test() function from the “stats” package), while Levene’s test (leveneTest() function from the “car” package) was used to assess homoscedasticity. To investigate the factors influencing the presence or absence of *Shewanella* species, two logistic regression models were tested, both assuming a binomial distribution for the dependent variable with a logit link function:

$$\mathrm{MODEL}\;1:\log\left(\frac{P(presence\;of\;Shewanella)}{1-P(presence\;of\;Shewanella)}\right)=\beta 0+Length+Weight+Sex+Month$$

$$\mathrm{MODEL}\;2:\log\left(\frac{P(presence\;of\;Shewanella)}{1-P(presence\;of\;Shewanella)}\right)=\beta 0+Length+Weight+Sex+Month+Temperature+Salinity+pH$$

where

- The fraction represents the log-odds of bacteria presence, i.e., the logarithm of the ratio between the probability of absence and the probability of presence of *Shewanella* spp. (binary dependent variable: 1 = presence, 0 = absence).
- “β0” is the intercept of the model, representing the reference value of the log-odds when all other variables are equal to zero.
- “Length” is the effect of length on the log-odds of *Shewanella* presence.
- “Weight” is the effect of weight on the log-odds of *Shewanella* presence.
- “Sex” (coded as a binary variable: 0 = male, 1 = female) is the effect of sex on the log-odds of *Shewanella* presence.
- “Month” (sampling month) represents the effect of each month on the log-odds of *Shewanella* presence compared to a reference month.
- “Temperature”, “Salinity”, and “pH” represent the monthly environmental conditions. Each physico-chemical variable captures the effect of the respective environmental factor on the log-odds of *Shewanella* presence.

To assess the collinearity among the independent variables in the model, the Variance Inflation Factor (VIF) was calculated (vif() function from the “car” package). Additionally, to examine the influence of each predictor on the dependent variable, a deviance analysis was performed with a Chi-square test (anova() with test = “Chisq”) to test the effect of each term added to the model. A stepwise regression approach (both directions) based on the Akaike Information Criterion (AIC) was applied, moreover, to the initial logistic model.

Also, to assess the stability and precision of the model, a bootstrap analysis was performed (boot() function from the “boot” package). The logistic regression model was applied to resampled data (1000 iterations), with the coefficients being extracted and the 95% confidence intervals calculated using the “boot.ci” function. These confidence intervals for each coefficient were derived from the bootstrap results, providing a robust measure of uncertainty in the estimates of the model. A statistical significance level of *p* < 0.05 was set.

## 3. Results

### 3.1. Blue Crab Morphology and Seawater Physico-Chemical Variables

The specimens analysed, considering the average across all the months (mean ± standard deviation), showed the following statistics: large males had a weight of 177.42 ± 65.66 g and a length of 133.38 ± 16.54 mm. Large females had a weight of 184.69 ± 73.59 g and a length of 138.82 ± 21.09 mm. Regarding the smaller specimens, males had a weight of 45.26 ± 14.38 g and a length of 87.10 ± 11.93 mm, while small females had a weight of 46.01 ± 14.97 g and a length of 87.83 ± 11.27 mm. In Table 1, the data for the specimens from which *Shewanella* species samples were isolated are presented.

The environmental data collected from June to October 2024 revealed fluctuations in temperature, salinity, and pH. Temperature ranged from 19.79 °C in October to 29.33 °C in August, with an average of 25.11 ± 3.81 °C. Salinity varied between 15.54 and 20.44, with an average of 18.55 ± 1.94, reaching its highest value in September at 20.44. pH levels remained relatively stable, ranging from 7.86 to 8.04, with a mean of 7.94 ± 0.07.

### 3.2. Prevalence and Factors Influencing Shewanella Species Presence in Blue Crab Haemolymph

**Figure 2:**
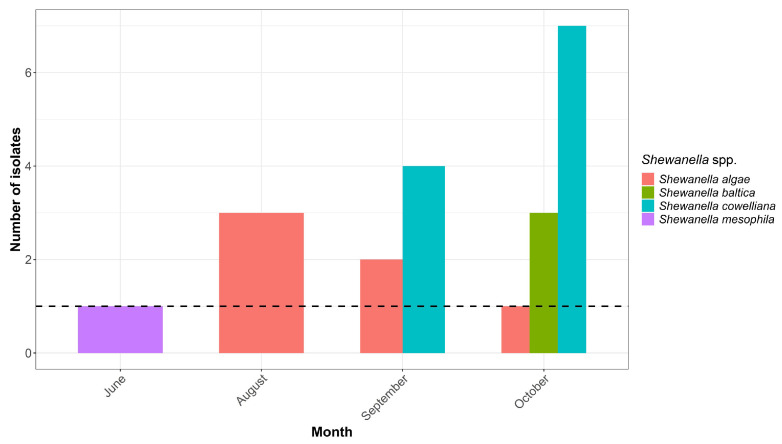
Number of *Shewanella* species isolates per month, with a dashed black line indicating the threshold (minimum value) of isolates. The plot highlights the monthly distribution and frequency of these species across the sampling period. July is excluded from the bar chart as no *Shewanella* isolates were detected.

The prevalence of *Shewanella* species, considering all isolates, in the blue crab haemolymph was found to be 4.75% (confidence interval (C.I.): 3.04–7.29%), with a total of 21 isolates from 300 specimens analysed (7%). Although 442 isolates were analysed, the number of unique samples was 300, as some specimens produced more than one isolate. Specifically, the prevalence of the different species followed this decreasing trend: *S. cowelliana* 2.49% (C.I.: 1.31–4.54%), *S. algae* 1.36% (C.I.: 0.55–3.08%), *S. mesophila* 0.23% (C.I.: 0.01–1.46%), and *S. baltica* 0.68% (C.I.: 0.18–2.14%) (Figure 2).

The results of the logistic regression models suggest that none of the variables analysed have a significant impact on the presence of the *Shewanella* genus. The variables “Length”, “Weight”, “Sex”, “Month” (with the categories June, September, and October), “Temperature”, “Salinity”, and “pH” are not significant predictors for the presence of these bacteria. The estimated coefficients for all variables are extremely low, with p-values equal to 1, indicating that these variables do not statistically significantly influence the probability of observing *Shewanella* species in the analysed sample. In particular, the intercept is very high but also not significant. Overall, the models demonstrate a poor fit to the data. The VIF analysis revealed strong multicollinearity for certain variables, with particularly high values for temperature (2230.71) and pH (2517.59), while length, weight, and salinity also showed elevated VIFs above 10. Furthermore, the deviance analysis showed no significant effects for any of the predictors, suggesting they do not contribute to explaining the variation in the presence or absence of *Shewanella* species in the models. Also, the stepwise regression analysis did not identify any significant predictors among the examined variables for the presence of *Shewanella* spp.

**Figure 3:**
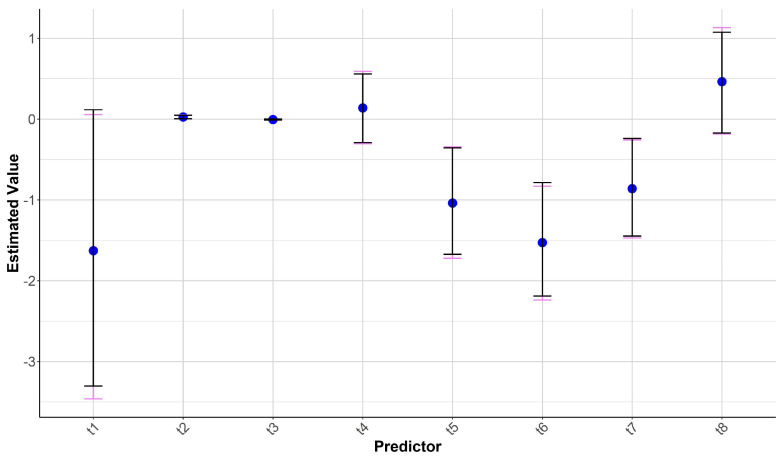
Bootstrap coefficients with 95% confidence intervals. The blue dots represent the estimated values based on the original data, while the violet error bars indicate the confidence intervals (C.I.s) based on standard errors (S.E.s). The black lines show the 95% bootstrap confidence intervals.

Based on the results and considering the variables involved, it was decided to proceed with Model 1 for further analysis. The non-parametric bootstrap analysis of Model 1 was performed to estimate the uncertainty of the coefficients in the logistic regression model with 1000 repetitions in order to obtain more robust estimates and calculate confidence intervals without making parametric assumptions (Figure 3). This approach allows for the consideration of data variability and provides a more reliable assessment of the coefficients, especially in the presence of uncertainty or poor statistical significance of the predictors in the logit model. Furthermore, the bootstrap allows for testing the stability of the estimates, identifying potential biases or large variations in the coefficients derived from the analysed samples. Therefore, this analysis complements the logit model, enhancing the robustness of the conclusions and increasing confidence in the obtained estimates.

In detail, the results of Model 1 showed that the estimated coefficients for the independent variables (length, weight, sex, and month) exhibited some degree of variability. In particular, the original estimate of the intercept (t1) was −1.594, with a standard error (S.E.) of 0.917. The 95% C.I. for this coefficient spanned from −3.457 to 0.126, suggesting the possibility of a non-significant effect for the intercept, as the interval includes zero.

The coefficient for the length variable (t2) was positive (0.026), with a precise estimate (SE: 0.011). The C.I. was between 0.004 and 0.049, indicating a positive relationship between length and the likelihood of the presence of total bacteria. This effect is likely to be statistically significant as the interval does not include zero. The coefficient for weight (t3) was negative (−0.005), with a small effect size and a low S.E. (0.004) and a C.I. ranging from −0.013 to 0.003, suggesting that weight might have a negligible or non-significant impact on the outcome. The coefficient for sex (t4) was positive (0.134), but with a larger S.E. (0.216) and a C.I. ranging from −0.292 to 0.574, indicating some imprecision in the estimate and suggesting that sex might not significantly influence the likelihood of bacteria presence. The coefficients for the months (t5-t8) varied from negative to positive values. Specifically, the coefficients for July (t5), June (t6), and October (t7) were negative (−1.014, −1.486, and −0.843, respectively), with the C.I.s suggesting significant negative effects. However, they had relatively high S.E.s (0.342, 0.370, and 0.306, respectively), indicating that the estimates for these months were less precise compared to other variables. The coefficient for September (t8) was positive (0.451), with a moderate S.E. (0.327), and its C.I. spanned from −0.157 to 1.093, suggesting a potential positive effect, though the interval includes zero, indicating that it may not be significant.

Overall, the biases for all coefficients were small, indicating that the model estimates were not significantly affected by the bootstrap procedure. However, the higher standard errors for some variables, such as months and sex, suggest that these coefficients may be less stable and more susceptible to variability in the data.

### 3.3. Antimicrobial Susceptibility Testing

**Figure 4:**
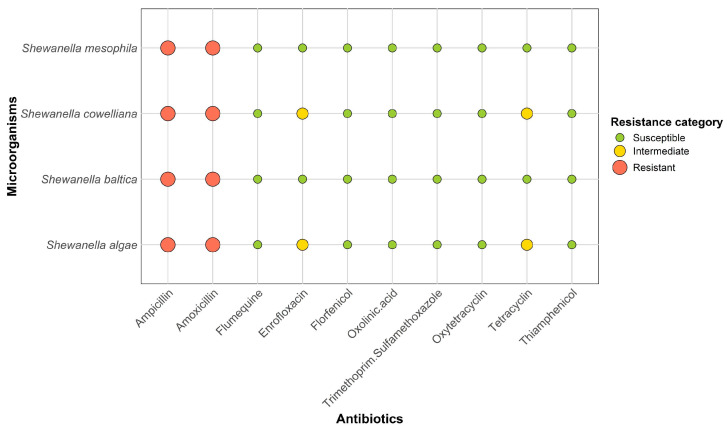
Antibiotic susceptibility patterns of *Shewanella* species isolated from blue crab haemolymph.

Figure 4 summarises the antibiotic resistance profiles of the *Shewanella* species isolated from blue crab haemolymph, selected based on their potential implications for human health as highlighted in the literature. The findings revealed similar resistance and susceptibility patterns across the species.

*Shewanella cowelliana*, *S. baltica*, *S. algae*, and *S. mesophila* are all resistant to ampicillin and amoxicillin. For the other antibiotics tested, sensitivity varies. *Shewanella cowelliana* and *S. algae* show intermediate resistance to enrofloxacin and tetracycline, while the other two species are sensitive. Furthermore, all species are sensitive to flumequine, florfenicol, oxolinic acid, trimethoprim/sulphamethoxazole, oxytetracycline, and thiamphenicol. Overall, resistance to the most commonly used classes of antibiotics is limited in these *Shewanella* species, except for ampicillin and amoxicillin.

### 3.4. Network Visualisation of Shewanella Species

The applied filter identified 114 articles published in English between 1996 and 2024. During the period from 1996 to 2006, scientific output was limited, averaging one paper per year, with two articles published in 1998 and 2005. From 2007 onwards, the number of publications showed an exponential increase, peaking in 2024 with 12 studies, the highest recorded within this timeframe.

**Figure 5:**
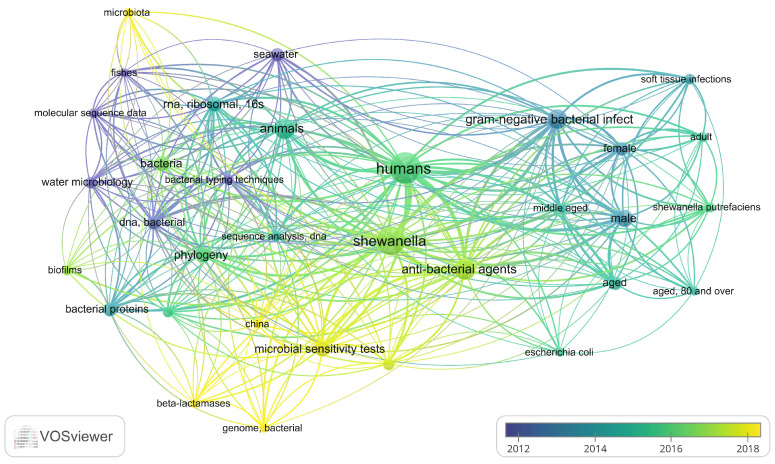
A bibliometric analysis of the *Shewanella* literature, showing keyword co-occurrence networks and highlighting associations between keywords. Thematic connections and emerging trends in the scientific literature are shown. Graph was generated using VOSviewer (v. 1.6.20) [47].

The network plot generated by VOSviewer highlights thematic clusters of keywords related to *Shewanella* research, illustrating the diverse areas of study, primarily focusing on *S. algae* and *S. putrefaciens*, species commonly associated with marine environments and human infections of varying severity (Figure 5). A central hub is formed around the keyword “*Shewanella*”, which connects various domains, reflecting its significance across multiple fields. The analysis reveals a strong focus on human health, with terms such as “humans”, “Gram-negative bacterial infections”, and “soft tissue infections” dominating one cluster, underscoring the clinical implications and pathogenic potential of *Shewanella* species, particularly *S. putrefaciens*. Another cluster emphasises environmental and ecological aspects, with keywords like “phylogeny”, “microbiota”, “biofilms”, and “water microbiology” pointing to the role of the genus in marine and aquatic environments. Additionally, molecular and antimicrobial studies are represented by terms such as “microbial sensitivity tests”, “beta-lactamases”, and “genome, bacterial”, reflecting research on resistance mechanisms and genomic characterisation. The overlap between clusters indicates interdisciplinary connections, particularly between environmental microbiology and human health, highlighting the broad relevance of *Shewanella* research.

## 4. Discussion

Among the more than 100 published studies, most focus primarily on two clinically significant Shewanellaceae species, *Shewanella putrefaciens* and *S. algae*, documenting cases of human infections with varying degrees of severity [18,28]. The first study, published in 1996, reports the initial cases of *S. algae* bacteraemia in patients with chronic leg ulcers, linked to a shared marine exposure during a warm summer, with varying clinical out-comes [48]. In 1998, a second study examined a co-infection caused by *S. putrefaciens* and *Mycobacterium marinum*, contracted at the beach [49]. Afterwards, other studies explored the presence of *S. algae* in European coastal waters and the impact of water temperature on its survival [50]. Between 1996 and 2006, the number of published studies remained relatively stable, averaging one publication per year. From 2008 onwards, scientific production increased, maintaining a steady rate of approximately two publications per year until 2011. In 2014, Diaz [51,52] published studies focusing on skin and soft tissue infections, as well as other infections resulting from marine injuries or exposures. More recently, Ibrahim et al. [53] conducted an analysis characterising potentially pathogenic strains of *S. algae* isolated from ballast water.

*Shewanella* infections caused by *S. algae* and *S. putrefaciens* generally present with clinical symptoms similar to those of other marine halophilic *Vibrio* species [54], although *S. putrefaciens* was identified in only 3% of clinical isolates, with no clear evidence of its pathogenic role [18].

In Denmark, *S. algae* is the primary cause of *Shewanella*-related ear infections, including both acute cases and exacerbations of chronic otitis media [18]. A study of 67 ear infections found *S. algae* responsible for all *Shewanella* cases, isolating it in 50% of the cases, while reports outside Denmark are rare and date back to the 1970s [18]. Vignier et al. [24] analysed 16 *Shewanella* human infections in Martinique and 239 global cases since 1997, involving soft tissue, ear, and abdominal infections, with an 87% recovery rate and 13% mortality. Skin and soft tissue infections, typically resulting from ulcers or trauma, are the most common manifestation, with bacteraemia being generally benign [18].

Two cases of *S. algae* bacteraemia, including one with myonecrosis in patients with chronic ulcers, have also been reported, along with primary bacteraemia linked to severe hepatobiliary disease and neoplasms, often progressing rapidly [55,56,57]. Also, *Shewanella* infections have been reported in 19 underweight neonates in South Africa, with bacteraemia linked to respiratory distress at birth, indicating intrapartum infection [18]. Respiratory colonisation and bacteraemia were also seen in patients with peritoneal dialysis, near-drowning, tuberculosis, and pleural empyema [18].

*Shewanella* species are commonly found in seawater with salinities of 15–20, particularly when temperatures exceed 13 °C [43], which may explain the increased incidence of infections in warmer climates or during hot summers in temperate regions [16]. Our findings from blue crab (*Callinectes sapidus*) haemolymph align with these environmental conditions, with observed temperatures ranging from 19.79 to 23.84 °C and salinities from 18.09 to 20.44.

Also, these environmental conditions may promote the production of potent neurotoxins by *Shewanella* species, such as tetrodotoxin and its analogues (TTXs) [58,59,60].

Several studies have demonstrated the ability of *Shewanella* species to produce TTXs, potentially contributing to clinical infections and ecological shifts. Some strains of *Shewanella* have been isolated from the intestines of food-poisoning patients and from contaminated food, with TTX production confirmed through mouse bioassays, suggesting that these bacteria can survive in the human gut and contribute to foodborne outbreaks [60]. In addition, *S. alga* was identified among TTX-producing bacteria isolated from marine organisms, further highlighting the ecological role of this genus in the marine environment [58]. Moreover, *S. putrefaciens* has been associated with TTX accumulation in pufferfish, including Lunartail puffer *Lagocephalus lunaris*, with its toxin production influenced by environmental conditions, particularly seawater temperature, indicating that ecological factors may regulate the toxigenic potential of *Shewanella* species [59].

Recently, advancements in pathogen identification and characterisation techniques have led to an increasing frequency of isolated strains from the Shewanellaceae family in aquatic organisms.

Regarding crustaceans, a multi-resistant *S. putrefaciens* isolate has been identified as the causative agent of hepato-pancreas necrosis in the Chinese mitten crab (*Eriocheir sinensis*), with an LD50 of 2.20 × 10^5^ CFU mL^−1^ [61].

A recent study also highlighted the presence of *S. algae* in blue crab from the coastal and lagoon areas of the northwestern Adriatic Sea [62]. In our study, we detected *S. algae* in the same geographical areas; however, there is a discrepancy between the number of specimens analysed and the detected percentages, with our results showing a lower prevalence (21/300, 7%) compared to those reported by Rubini et al. [62] (18/72, 25%). These differences in percentages could be due to differences in the number of specimens analysed, sampling methods, diagnostic techniques, or geographical areas examined, all of which may influence the detection of *S. algae*. Conversely, our results are similar to those found by Richards et al. [63], where from 276 water and shellfish samples analysed, 1,421 bacterial isolates were selected, with 170 (12.0%) identified as *Shewanella* spp., the majority being *S. putrefaciens*. However, our study is a preliminary investigation. It would be beneficial to improve the models by extending the sampling period to better capture the prevalence of *Shewanella* species throughout the year. The low number of isolates (21) limits the ability to achieve statistical significance, and the high correlation between temperature, salinity, pH, and month may hinder the capacity of the models to differentiate their individual effects. Increasing the sample size and refining the model would provide a more accurate understanding of the variables influencing the presence of *Shewanella* species in the blue crab haemolymph.

Several *Shewanella* species have also been isolated from both freshwater and marine fishes. For example, *S. putrefaciens* was first isolated from diseased Marbled spinefoot (*Siganus rivulatus*) in 1985 [64], with few reports of fish infections. *Shewanella putrefaciens* was isolated from common carp (*Cyprinus carpio*) and rainbow trout (*Oncorhynchus mykiss*) [65], while *S. algae* strains were isolated in 2011 from Italian aquaculture farms along the northern and southern Adriatic Sea, specifically from European seabass (*Dicentrarchus labrax*), gilthead seabream (*Sparus aurata*), and shi drum (*Umbrina cirrosa*) [66] (Table S1). The study of Zago et al. [67] identified beta-lactam resistance genes in marine strains from Italian aquaculture [66]. Multidrug-resistant (MDR) *S. algae* clones were traced along the Adriatic coast, showing point mutations and phylogenetic links [67]. The rise of community-acquired infections caused by antibiotic-resistant bacteria has highlighted the presence of antimicrobial resistance genes (ARGs) in natural environments, including seawater. Resistance to beta-lactams, widely used in medicine, has significantly increased [68,69]. However, limited data are available in Italy on the prevalence of ARGs in emerging pathogens, such as *Shewanella* species, which are responsible for human infections. Most studies focus on resistance to beta-lactams like penicillins and cephalosporins [70] and other antibiotics such as tetracycline and sulphonamides [66]. A recent study by Torri et al. [71] reported *S. algae* infections showing susceptibility to third-generation cephalosporins and gentamicin, while amikacin, carbapenems, and piperacillin/tazobactam proved effective. These bacteria are emerging as MDR pathogens, harbouring resistance genes to beta-lactams and quinolones, often carried by mobile genetic elements [16]. Findings have highlighted the role of *Shewanella* in spreading antibiotic resistance, particularly through plasmids, transposons, and integrons [16]. Resistance mechanisms include beta-lactamases, altered penicillin-binding proteins (PBPs), and efflux systems, with countermeasures including stable beta-lactams and agents targeting resistant PBPs [68]. Additionally, beta-lactam antibiotics degrade under environmental conditions, with degradation rates influenced by pH, temperature, and functional group reactivity, leading to reduced antimicrobial activity [72]. Pangenome analysis of *S. xiamenensis* has also revealed genetic traits associated with its diversity, pathogenicity, and evolving antibiotic resistance, highlighting recent changes in its resistance spectrum due to acquired genes [17]. Our results align with the current literature, confirming the trends of beta-lactam antibiotic resistance observed in previous studies.

## 5. Conclusions

*Shewanella* species are environmental bacteria commonly found in marine and coastal environments. Previous studies have highlighted the widespread presence of these bacteria in both seawater and aquatic organisms, such as fish, crustaceans, and bivalve molluscs. A significant presence of potentially harmful *Shewanella* strains has also been identified in the marine coastal waters of the Adriatic Sea in Italy, with occasional human infections reported between 2013 and 2016, primarily caused by *S. algae*. These infections are strongly associated with marine water exposure during the warmer months.

Climate change, with rising temperatures and salinity levels, may further facilitate the spread and persistence of these pathogens, increasing the likelihood of contact with both aquatic organisms and humans year-round. However, proper cooking of blue crabs and other aquatic species eliminates the risk to consumers. The risk may increase during handling, especially in aquatic environments without proper personal protective equipment (PPE; e.g., gloves), as this can expose individuals to the bacteria. The most vulnerable individuals are immunocompromised patients and elderly people (over 60 years old), with severe cases or fatalities being rare and often linked to other complications. In contrast, children (aged 9 years and under) tend to experience mild symptoms, such as otitis, which is not a cause for concern.

Further research is needed to better understand the presence of *Shewanella* during different months and its transmission through aquatic species such as blue crabs. Increasing the sample size in future studies is therefore recommended to enhance detection power. Moreover, this study highlights the need to verify the virulence of these motile Gram-negative bacilli, as the current study does not provide conclusive evidence of their pathogenicity. Accordingly, future investigations are required to assess the potential risks posed by these bacteria and their role in disease transmission. A One Health approach, integrating human and veterinary medicine, will be crucial in addressing this issue. Additionally, the growing concern regarding antibiotic resistance in this pathogen, which may lead to more severe clinical complications, underscores the importance of continued investigation, particularly in the context of the increasing prevalence of resistant strains.

## Figures and Tables

**Table 1:** Morphometric characteristics of blue crabs (length in mm, weight in g) and identification of *Shewanella* species, including MALDI-TOF MS log-scores (protein profile similarity) and sequence match percentages (GenBank reference data).

Specimens	Size	Length	Weight	Sex	Month	MALDI-TOF MS	Log-Score	Molecular Analysis	% Match	Bacteria
4	Large	188	364.98	Female	June	*Shewanella mesophila*	2.27	*Shewanella mesophila*	0	*S. mesophila*
11	Large	114	124.75	Female	August	*Shewanella algae*	2.35	0	0	*S. algae*
1	Small	96	69.37	Female	August	*S. algae*	2.36	0	0	*S. algae*
20	Small	95	53.99	Male	August	*S. algae*	2.36	0	0	*S. algae*
6	Large	119	205.74	Male	September	*S. algae*	2.36	0	0	*S. algae*
20	Large	137	141.77	Female	September	*S. algae*	2.31	0	0	*S. algae*
22	Large	110	113.04	Male	September	*Shewanella cowelliana*	2.34	0	0	*S. cowelliana*
30	Large	135	139.97	Female	September	*S. cowelliana*	2.36	0	0	*S. cowelliana*
16	Small	56	11.34	Female	September	*S. cowelliana*	2.28	0	0	*S. cowelliana*
21	Small	88	45.00	Female	September	*S. cowelliana*	2.12	0	0	*S. cowelliana*
2	Large	154	203.22	Male	October	*S. cowelliana*	2.13	0	0	*S. cowelliana*
3	Large	133	156.86	Male	October	*S. cowelliana*	2.23	0	0	*S. cowelliana*
15	Large	133	157.89	Female	October	*Shewanella* spp.	1.78	*S. baltica*	99.58	*S. baltica*
16	Large	132	155.67	Male	October	*S. cowelliana*	2.24	0	0	*S. cowelliana*
18	Large	130	151.45	Male	October	*Shewanella baltica*	2.32	0	0	*S. baltica*
8	Small	91	57.88	Female	October	*Shewanella* spp.	1.95	*S. baltica*	99.58	*S. baltica*
18	Small	99	63.89	Female	October	*S. algae*	2.36	0	0	*S. algae*
18	Small	99	63.89	Female	October	*S. cowelliana*	2.35	0	0	*S. cowelliana*
19	Small	91	58.32	Female	October	*S. cowelliana*	2.34	0	0	*S. cowelliana*
21	Small	99	65.78	Female	October	*S. cowelliana*	2.33	0	0	*S. cowelliana*
23	Small	98	63.87	Male	October	*S. cowelliana*	2.32	0	0	*S. cowelliana*

## Data Availability

All data generated or analysed during this study are included in this published article and its Supplementary Materials.

## Supplementary Materials

The following supporting information can be downloaded at https://www.mdpi.com/article/10.3390/ani15121731/s1, Table S1. Main *Shewanella* species isolated from aquatic organisms and associated animal diseases, with only *Shewanella algae*, *Shewanella putrefaciens*, and *Shewanella haliotis* linked to human infections [59,61,62,64,65,66,73,74,75,76,77,78,79,80,81,82,83,84,85,86,87,88,89,90]; Figure S1. Annual trends of temperature and salinity. Black dots indicate peak values, while the months during which sampling occurred are marked in green on the X-axis.

## References

[1] Galil B.S., Froglia C., Noel P., Briand F. (2002). CIESM Atlas of Exotic Species in the Mediterranean: Decapods and Stomatopods.

[2] Piras P., Esposito G., Meloni D. (2019). On the Occurrence of the Blue Crab *Callinectes Sapidus* (Rathbun, 1896) in Sardinian Coastal Habitats (Italy): A Present Threat or a Future Resource for the Regional Fishery Sector?. BioInvasions Rec..

[3] Mancinelli G., Bardelli R., Zenetos A. (2021). A Global Occurrence Database of the Atlantic Blue Crab *Callinectes Sapidus*. Sci. Data.

[4] Marchessaux G., Mangano M.C., Bizzarri S., M’Rabet C., Principato E., Lago N., Veyssiere D., Garrido M., Scyphers S.B., Sarà G. (2023). Invasive Blue Crabs and Small-Scale Fisheries in the Mediterranean Sea: Local Ecological Knowledge, Impacts and Future Management. Mar. Policy.

[5] Azzurro E., Bonanomi S., Chiappi M., De Marco R., Luna G.M., Cella M., Guicciardi S., Tiralongo F., Bonifazi A., Strafella P. (2024). Uncovering Unmet Demand and Key Insights for the Invasive Blue Crab (*Callinectes Sapidus*) Market before and after the Italian Outbreak: Implications for Policymakers and Industry Stakeholders. Mar. Policy.

[6] Shields J.D., Overstreet R.M. (2003). The Blue Crab: Diseases, Parasites and Other Symbionts. Fac. Publ. Harold W. Manter Lab. Parasitol..

[7] Davis J.W., Sizemore R.K. (1982). Incidence of Vibrio Species Associated with Blue Crabs (*Callinectes Sapidus*) Collected from Galveston Bay, Texas. Appl. Environ. Microbiol..

[8] Welsh P.C., Sizemore R.K. (1985). Incidence of Bacteremia in Stressed and Unstressed Populations of the Blue Crab, *Callinectes Sapidus*. Appl. Environ. Microbiol..

[9] Johnson N.G., Burnett L.E., Burnett K.G. (2011). Properties of Bacteria That Trigger Hemocytopenia in the Atlantic Blue Crab, *Callinectes Sapidus*. Biol. Bull..

[10] Rippey S.R. (1994). Infectious Diseases Associated with Molluscan Shellfish Consumption. Clin. Microbiol. Rev..

[11] Farag M.A., Mansour S.T., Nouh R.A., Khattab A.R. (2023). Crustaceans (Shrimp, Crab, and Lobster): A Comprehensive Review of Their Potential Health Hazards and Detection Methods to Assure Their Biosafety. J. Food Saf..

[12] Koutsoumanis K., Allende A., Alvarez-Ordóñez A., Bolton D., Bover-Cid S., Chemaly M., De Cesare A., Herman L., Hilbert F., EFSA Panel on Biological Hazards (BIOHAZ) (2024). Public Health Aspects of Vibrio Spp. Related to the Consumption of Seafood in the EU. EFSA J..

[13] Weiskopf S.R., Rubenstein M.A., Crozier L.G., Gaichas S., Griffis R., Halofsky J.E., Hyde K.J.W., Morelli T.L., Morisette J.T., Muñoz R.C. (2020). Climate Change Effects on Biodiversity, Ecosystems, Ecosystem Services, and Natural Resource Management in the United States. Sci. Total Environ..

[14] IPCC Intergovernmental Panel on Climate Change (2022). Climate Change: A Threat to Human Wellbeing and Health of the Planet. Taking Action Now Can Secure Our Future, IPCC Reports.

[15] Kouzuma A., Kasai T., Hirose A., Watanabe K. (2015). Catabolic and Regulatory Systems in *Shewanella oneidensis* MR-1 Involved in Electricity Generation in Microbial Fuel Cells. Front. Microbiol..

[16] Yousfi K., Bekal S., Usongo V., Touati A. (2017). Current Trends of Human Infections and Antibiotic Resistance of the Genus *Shewanella*. Eur. J. Clin. Microbiol. Infect. Dis..

[17] Wang H., Xia F., Xia Y., Li J., Hu Y., Deng Y., Zou M. (2024). Pangenome Analysis of *Shewanella xiamenensis* Revealed Important Genetic Traits Concerning Genetic Diversity, Pathogenicity and Antibiotic Resistance. BMC Genom..

[18] Holt H.M., Gahrn-Hansen B., Bruun B. (2005). Shewanella Algae and *Shewanella putrefaciens*: Clinical and Microbiological Characteristics. Clin. Microbiol. Infect..

[19] Cramer W., Guiot J., Marini K., MedECC (2020). Climate and Environmental Change in the Mediterranean Basin—Current Situation and Risks for the Future.

[20] Palevich N., Palevich F.P., Gardner A., Brightwell G., Mills J. (2022). Genome Collection of *Shewanella* Spp. Isolated from Spoiled Lamb. Front. Microbiol..

[21] Lemaire O.N., Méjean V., Iobbi-Nivol C. (2020). The *Shewanella* Genus: Ubiquitous Organisms Sustaining and Preserving Aquatic Ecosystems. FEMS Microbiol. Rev..

[22] NCBI National Center for Biotechnology Information Taxonomy Browser (Shewanella). https://www.ncbi.nlm.nih.gov/Taxonomy/Browser/wwwtax.cgi?mode=Info&id=22.

[23] Hau H.H., Gralnick J.A. (2007). Ecology and Biotechnology of the Genus *Shewanella*. Annu. Rev. Microbiol..

[24] Vignier N., Barreau M., Olive C., Baubion E., Théodose R., Hochedez P., Cabié A. (2013). Human Infection with *Shewanella putrefaciens* and *S. Algae*: Report of 16 Cases in Martinique and Review of the Literature. Am. Soc. Trop. Med. Hyg..

[25] Zong Z. (2011). Nosocomial Peripancreatic Infection Associated with *Shewanella xiamenensis*. J. Med. Microbiol..

[26] Antonelli A., Di Palo D.M., Galano A., Becciani S., Montagnani C., Pecile P., Galli L., Rossolini G.M. (2015). Intestinal Carriage of *Shewanella Xiamenensis* Simulating Carriage of OXA-48–Producing Enterobacteriaceae. Diagn. Microbiol. Infect. Dis..

[27] Chia-Wei L., Cheng J.-F., Tung K.-C., Hong Y.-K., Lin J.-H., Lin Y.-H., Tsai C.-A., Lin S.-P., Chen Y.-C., Shi Z.-Y. (2022). Evolution of Trimethoprim/Sulfamethoxazole Resistance in *Shewanella algae* from the Perspective of Comparative Genomics and Global Phylogenic Analysis. J. Microbiol. Immunol. Infect..

[28] Yu K., Huang Z., Xiao Y., Wang D. (2022). *Shewanella* Infection in Humans: Epidemiology, Clinical Features and Pathogenicity. Virulence.

[29] Yiallouros P., Mavri A., Attilakos A., Moustaki M., Leontsini F., Karpathios T. (2013). *Shewanella putrefaciens* Bacteraemia Associated with Terminal Ileitis. Paediatr. Int. Child Health.

[30] Dey S., Bhattacharya D., Roy S., Nadgir S., Patil A., Kholkute S. (2015). *Shewanella algae* in Acute Gastroenteritis. Indian J. Med. Microbiol..

[31] Fang Y., Wang Y., Liu Z., Lu B., Dai H., Kan B., Wang D. (2017). *Shewanella Carassii* Sp. Nov., Isolated from Surface Swabs of Crucian Carp and Faeces of a Diarrhoea Patient. Int. J. Syst. Evol. Microbiol..

[32] Liu D., Wilson C., Hearlson J., Singleton J., Thomas R.B., Crupper S.S. (2013). Prevalence of Antibiotic-Resistant Gram-Negative Bacteria Associated with the Red-Eared Slider (Trachemys Scripta Elegans). J Zoo Wildl. Med..

[33] Yarza P., Yilmaz P., Pruesse E., Glöckner F.O., Ludwig W., Schleifer K.-H., Whitman W.B., Euzéby J., Amann R., Rosselló-Móra R. (2014). Uniting the Classification of Cultured and Uncultured Bacteria and Archaea Using 16S rRNA Gene Sequences. Nat. Rev. Microbiol..

[34] Fang Y., Wang Y., Liu Z., Dai H., Cai H., Li Z., Du Z., Wang X., Jing H., Wei Q. (2019). Multilocus Sequence Analysis, a Rapid and Accurate Tool for Taxonomic Classification, Evolutionary Relationship Determination, and Population Biology Studies of the Genus *Shewanella*. Appl. Environ. Microbiol..

[35] Thorell K., Meier-Kolthoff J.P., Sjöling Å., Martín-Rodríguez A.J. (2019). Whole-Genome Sequencing Redefines *Shewanella* Taxonomy. Front. Microbiol..

[36] Lee I., Ouk Kim Y., Park S.-C., Chun J. (2016). OrthoANI: An Improved Algorithm and Software for Calculating Average Nucleotide Identity. Int. J. Syst. Evol. Microbiol..

[37] Chiesa S., Petochi T., Brusà R.B., Raicevich S., Cacciatore F., Franceschini G., Antonini C., Vallini C., Bernarello V., Oselladore F. (2025). Impacts of the Blue Crab Invasion on Manila Clam Aquaculture in Po Delta Coastal Lagoons (Northern Adriatic Sea, Italy). Estuar. Coast. Shelf Sci..

[38] Italy Decreto Legislativo 4 Marzo 2014, n. 26. Attuazione Della Direttiva 2010/63/UE Sulla Protezione Degli Animali Utilizzati a Fini Scientifici. https://www.Gazzettaufficiale.It/Eli/Id/2014/03/14/14G00036/Sg.

[39] European Parliament and Council of the European Union Directive 2010/63/EU of the European Parliament and of the Council of 22 September 2010 on the Protection of Animals Used for Scientific Purposes. https://eur-lex.europa.eu/eli/dir/2010/63/oj/eng.

[40] European Parliament and Council of the European Union Regulation (EU) No. 1143/2014 of the European Parliament and of the Council of 22 October 2014 on the Prevention and Management of the Introduction and Spread of Invasive Alien Species. https://eur-lex.europa.eu/eli/reg/2014/1143/oj/eng.

[41] Italy Decreto Legislativo 15 Dicembre 2017, n. 230. Adeguamento Della Normativa Nazionale Alle Disposizioni Del Regolamento (UE) n. 1143/2014 Del Parlamento Europeo e Del Consiglio Del 22 Ottobre 2014. https://Www.Gazzettaufficiale.It/Eli/Id/2018/01/30/18G00012/Sg.

[42] Noga E.J. (2010). Fish Disease: Diagnosis and Treatment.

[43] Clinical and Laboratory Standards Institute (CLSI) (2020). Methods for Antimicrobial Broth Dilution and Disk Diffusion Susceptibility Testing of Bacteria Isolated from Aquatic Animals. CLSI Guideline VET03.

[44] Clinical and Laboratory Standards Institute (CLSI) (2020). Performance Standards for Antimicrobial Susceptibility Testing of Bacteria Isolated from Aquatic Animals. CLSI Supplement VET04.

[45] Pastorino P., Colussi S., Pizzul E., Varello K., Menconi V., Mugetti D., Tomasoni M., Esposito G., Bertoli M., Bozzetta E. (2021). The Unusual Isolation of Carnobacteria in Eyes of Healthy Salmonids in High-Mountain Lakes. Sci. Rep..

[46] Ki J.-S., Zhang W., Qian P.-Y. (2009). Discovery of Marine Bacillus Species by 16S rRNA and rpoB Comparisons and Their Usefulness for Species Identification. J. Microbiol. Methods.

[47] Van Eck N., Waltman L. (2010). Software Survey: VOSviewer, a Computer Program for Bibliometric Mapping. Scientometrics.

[48] Dominguez H., Vogel B.F., Gram L., Hoffmann S., Schaebel S. (1996). *Shewanella alga* Bacteremia in Two Patients with Lower Leg Ulcers. Clin. Infect. Dis..

[49] Papanaoum K., Marshmann G., Gordon L.A., Lumb R., Gordon D.L. (1998). Concurrent Infection Due to *Shewanella putrefaciens and Mycobacterium Marinum* Acquired at the Beach. Australas. J. Dermatol..

[50] Gram L., Bundvad A., Melchiorsen J., Johansen C., Fonnesbech Vogel B. (1999). Occurrence of *Shewanella algae* in Danish Coastal Water and Effects of Water Temperature and Culture Conditions on Its Survival. Appl. Environ. Microbiol..

[51] Diaz J.H. (2014). Skin and Soft Tissue Infections Following Marine Injuries and Exposures in Travelers. J. Travel Med..

[52] Diaz J.H. (2014). Emerging Causes of Superficial and Invasive Infections Following Marine Injuries and Exposures. J. La. State Med. Soc. Off. Organ La. State Med. Soc..

[53] Ibrahim N.N.N., Nasir N.M., Sahrani F.K., Ahmad A., Sairi F. (2021). Characterization of Putative Pathogenic *Shewanella algae* Isolated from Ballast Water. Vet. World.

[54] Dalsgaard A., Frimodt-Møller N., Bruun B., Høi L., Larsen J.L. (1996). Clinical Manifestations and Molecular Epidemiology of *Vibrio vulnificus* Infections in Denmark. Eur. J. Clin. Microbiol. Infect. Dis..

[55] Brink A.J., Van Straten A., Van Rensburg A.J. (1995). *Shewanella* (*Pseudomonas*) *putrefaciens* Bacteremia. Clin. Infect. Dis..

[56] Chen Y., Liu Y., Yen M., Wang J., Wang J., Wann S., Cheng D. (1997). Skin and Soft-Tissue Manifestations of *Shewanella putrefaciens* Infection. Clin. Infect. Dis..

[57] Kim J.H., Cooper R.A., Welty-Wolf K.E., Harrell L.J., Zwadyk P., Klotman M.E. (1989). *Pseudomonas putrejaciens* Bacteremia. Clin. Infect. Dis..

[58] Simidu U., Kita-Tsukamoto K., Yasumoto T., Yotsu M. (1990). Taxonomy of Four Marine Bacterial Strains that Produce Tetrodotoxin. Int. J. Syst. Bacteriol..

[59] Auawithoothij W., Noomhorm A. (2012). *Shewanella putrefaciens*, a Major Microbial Species Related to Tetrodotoxin (TTX)-Accumulation of Puffer Fish *Lagocephalus lunaris*. J. Appl. Microbiol..

[60] Wang D., Wang Y., Huang H., Lin J., Xiao D., Kan B. (2013). Identification of Tetrodotoxin-Producing *Shewanella* Spp. from Feces of Food Poisoning Patients and Food Samples. Gut Pathog..

[61] Liu J., Teng C., Zheng X., Xu L., Cao H., Gai C. (2024). *Shewanella putrefaciens* as an Emerging Pathogen of Hepatopancreas Necrosis Disease in Chinese Mitten Crab *Eriocheir sinensis*. Dis. Aquat. Organ..

[62] Rubini S., Massella E., Bertasi B., Mangeri L., Dell’Orfano G., Russo S., Sampieri M., Tiralongo F., Taddei R. (2024). Isolation of *Shewanella algae* from Blue Crabs (*Callinectes sapidus*) in Northwestern Adriatic Sea. Eur. J. Public Health.

[63] Richards G.P., Watson M.A., Crane E.J., Burt I.G., Bushek D. (2008). *Shewanella* and *Photobacterium* Spp. in Oysters and Seawater from the Delaware Bay. Appl. Environ. Microbiol..

[64] Saeed M., Alamoudi M., Al-Harbi A. (1987). A Pseudomonas Associated with Disease in Cultured Rabbitfish *Siganus rivulatus* in the Red Sea. Dis. Aquat. Organ..

[65] Kozinska A., Pekala A. (2004). First Isolation of *Shewanella putrefaciens* from Freshwater Fish-a Potential New Pathogen of Fish. Bull.-Eur. Assoc. Fish Pathol..

[66] Labella A., Gennari M., Ghidini V., Trento I., Manfrin A., Borrego J.J., Lleo M.M. (2013). High Incidence of Antibiotic Multi-Resistant Bacteria in Coastal Areas Dedicated to Fish Farming. Mar. Pollut. Bull..

[67] Zago V., Veschetti L., Patuzzo C., Malerba G., Lleo M.M. (2020). *Shewanella algae* and *Vibrio* Spp. Strains Isolated in Italian Aquaculture Farms are Reservoirs of Antibiotic Resistant Genes That Might Constitute a Risk for Human Health. Mar. Pollut. Bull..

[68] Poole K. (2004). Resistance to β-Lactam Antibiotics. Cell. Mol. Life Sci..

[69] Bush K., Bradford P.A. (2016). β-Lactams and β-Lactamase Inhibitors: An Overview. Cold Spring Harb. Perspect. Med..

[70] Ottaviani D., Leoni F., Talevi G., Masini L., Santarelli S., Rocchegiani E., Susini F., Montagna C., Monno R., D’Annibale L. (2013). Extensive Investigation of Antimicrobial Resistance in Vibrio Parahaemolyticus from Shellfish and Clinical Sources, Italy. Int. J. Antimicrob. Agents.

[71] Torri A., Bertini S., Schiavone P., Congestrì F., Matteucci M., Sparacino M., Testa G., Pedna M.F., Sambri V. (2018). *Shewanella algae* Infection in Italy: Report of 3 Years’ Evaluation along the Coast of the Northern Adriatic Sea. New Microbes New Infect..

[72] Mitchell S.M., Ullman J.L., Teel A.L., Watts R.J. (2014). pH and Temperature Effects on the Hydrolysis of Three β-Lactam Antibiotics: Ampicillin, Cefalotin and Cefoxitin. Sci. Total Environ..

[73] Hou D., Lian T., Guo G., Gong H., Wu C., Han P., Weng S., He J. (2023). Integration of Microbiome and Koch’s Postulates to Reveal Multiple Bacterial Pathogens of Whitish Muscle Syndrome in Mud Crab, *Scylla paramamosain*. Microbiome.

[74] Venmathi Maran B.A., Iwamoto E., Okuda J., Matsuda S., Taniyama S., Shida Y., Asakawa M., Ohtsuka S., Nakai T., Boxshall G.A. (2007). Isolation and Characterization of Bacteria from the Copepod Pseudocaligus Fugu Ectoparasitic on the Panther Puffer *Takifugu pardalis* with the Emphasis on TTX. Toxicon.

[75] Migaou M., Macé S., Maalej H., Marchand L., Bonnetot S., Noël C., Sinquin C., Jérôme M., Zykwinska A., Colliec-Jouault S. (2024). Exploring the Exopolysaccharide Production Potential of Bacterial Strains Isolated from Tunisian Blue Crab Portunus Segnis Microbiota. Molecules.

[76] Ariole C.N., Eddo T.T. (2016). Effect of an Indigenous Probiotic (*Shewanella algae*) Isolated from Healthy Shrimp (*Penaeus Monodon*) Intestine on *Clarias gariepinus*. Int. J. Aquac..

[77] Cao H., Chen S., Lu L., An J. (2018). *Shewanella algae*: An Emerging Pathogen of Black Spot Disease in Freshwater-Cultured Whiteleg Shrimp (*Penaeus vannamei*). Isr. J. Aquac. Bamidgeh.

[78] Villarias M.P., Gabuat H.G., Romey M.T., Pakingking R., Ynion G.P.L., Fagutao F., Suharman I., Caipang C.M. (2024). Antibiotic Resistance and Molecular Identification of Dominant Bacteria Associated with Loose Shell Syndrome in Mangrove Crabs (*Scylla* Spp.). BIO Web Conf..

[79] Chistoserdov A.Y., Smolowitz R., Mirasol F., Hsu A. (2005). Culture-Dependent Characterization of the Microbial Community Associated with Epizootic Shell Disease Lesions in American Lobster, Homarus Americanus. J. Shellfish Res..

[80] Lailaja V.P., Sumithra T.G., Reshma K.J., Anusree V.N., Amala P.V., Kishor T.G., Sanil N.K. (2022). Characterization of Novel L-Asparaginases Having Clinically Safe Profiles from Bacteria Inhabiting the Hemolymph of the Crab, *Scylla serrata* (Forskål, 1775). Folia Microbiol..

[81] Faizul M.I.M., Eng H.T., Christianus A., Abdel-Hadi Y.M., Carmichael R.H., Botton M.L., Shin P.K.S., Cheung S.G. (2015). Bacteria and Fungi Identified on Horseshoe Crabs, *Tachypleus gigas* and *Carcinoscorpius rotundicauda* in the Laboratory. Changing Global Perspectives on Horseshoe Crab Biology, Conservation and Management.

[82] Lee Y.-H., Tung K.-C., Cheng J.-F., Wu Z.-Y., Chen S.-Y., Hong Y.-K., Huang Y.-T., Liu P.-Y. (2019). Genomic Characterization of Carbapenem-Resistant *Shewanella algae* Isolated from Asian Hard Clam (*Meretrix lusoria*). Aquaculture.

[83] Ratnawati S.E., Kuuliala L., Verschuere N., Cnockaert M., Vandamme P., Devlieghere F. (2024). The Exploration of Dominant Spoilage Bacteria in Blue Mussels (*Mytilus edulis*) Stored under Different Modified Atmospheres by MALDI-TOF MS in Combination with 16S rRNA Sequencing. Food Microbiol..

[84] Ivanova E.P., Sawabe T., Gorshkova N.M., Svetashev V.I., Mikhailov V.V., Nicolau D.V., Christen R. (2001). *Shewanella japonica* Sp. Nov. Int. J. Syst. Evol. Microbiol..

[85] Wu Z.-Y., Liu P.-Y., Tseng S.-Y., Lee Y.-H., Ho S.-P. (2018). Characteristics and Phylogeny of *Shewanella haliotis* Isolated from Cultivated Shellfish in Taiwan. Can. J. Infect. Dis. Med. Microbiol..

[86] Farto R., Guisande J.A., Armada S.P., Prado S., Nieto P. (2006). An Improved and Rapid Biochemical Identification of Indigenous Aerobic Culturable Bacteria Associated with Galician Oyster Production. J. Shellfish Res..

[87] Beleneva I.A., Magarlamov T.Y., Eliseikina M.G., Zhukova N.V. (2009). Biochemical and Pathogenic Properties of the Natural Isolate of *Shewanella algae* from Peter the Great Bay, Sea of Japan. J. Invertebr. Pathol..

[88] Leonardo M.R., Moser D.P., Barbieri E., Brantner C.A., MacGregor B.J., Paster B.J., Stackebrandt E., Nealson K.H. (1999). *Shewanella pealeana* Sp. Nov., a Member of the Microbial Community Associated with the Accessory Nidamental Gland of the Squid *Loligo pealei*. Int. J. Syst. Evol. Microbiol..

[89] Matsui T., Taketsugu S., Sato H., Yamamori K., Kodama K., Ishii A., Hirose H., Shimizu C. (1990). Toxification of Cultured Puffer Fish by the Administration of Tetrodotoxin Producing Bacteria. Nippon. Suisan Gakkaishi.

[90] Korun J., Akgun-Dar K., Yazici M. (2009). Isolation of *Shewanella putrefaciens* from Cultured European Sea Bass, (*Dicentrarchus labrax*) in Turkey. Rev. Med. Vet..

