# Supplementary figures and images for: Blue Crab (Callinectes sapidus) Haemolymph as a Potential Reservoir of Mesophilic Shewanella Species
Source: Animals (Basel). 2025 Jun 11;15(12):1731. doi: 10.3390/ani15121731 (PMC12189129; doi:10.3390/ani15121731)

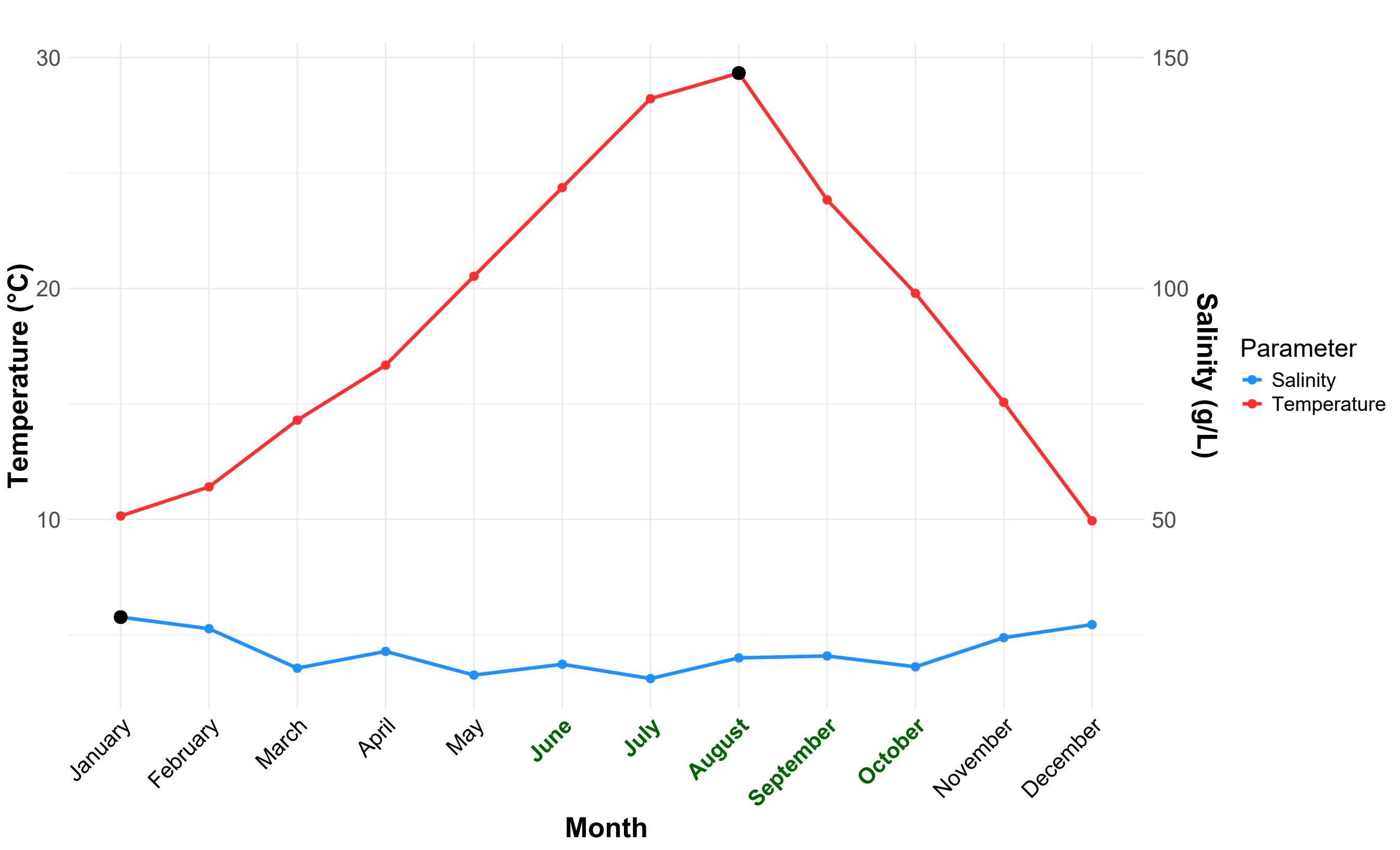

Supplement: Supplementary file 1 [file animals-15-01731-s001.zip › Figure S1.pdf]
